# Assessing patient satisfaction and healthcare delivery amidst the COVID-19 pandemic: insights from Jammu and Kashmir, India

**DOI:** 10.1186/s12889-024-18986-w

**Published:** 2024-08-01

**Authors:** Arunima Koul, Shazina Saeed, Karuna Nidhi Kaur, Farah Niazi

**Affiliations:** 1 Arunima Koul, Laboratory of Disease Dynamics & Molecular Epidemiology, Amity Institute of Public Health, Amity University, Noida, India; 2 Amity Institute of Public Health, Amity University, Noida, India; 3 Laboratory of Disease Dynamics & Molecular Epidemiology, Amity Institute of Public Health, Karuna Nidhi Kaur, Amity University, Noida, India

**Keywords:** Patient satisfaction, COVID-19, Healthcare delivery, Quality of healthcare, Jammu and Kashmir

## Abstract

**Introduction:**

Amidst the challenges posed by Covid-19, assessing healthcare quality in India is crucial, particularly through patient satisfaction levels.

**Methodology:**

A cross-sectional survey of 277 participants in Jammu and Kashmir was conducted, utilizing a semi-structured questionnaire and PSQ-18. Data analysis was performed using SPSS (v25) including Chi-Square tests and Descriptive analysis.

**Results:**

Out of 277 participants, 70.8% expressed high satisfaction with medical care. Majority (70%) agreed that doctors explained medical tests well. Additionally, 70% strongly agreed that their doctor’s office was well-equipped. Dissatisfaction factors were notably low. Significant associations were found between age and alcohol use (*p* = 0.041), gender and alcohol use (*p* = 0.007), gender and tobacco use (*p* = 0.032), and education level and vaccination (*p* = 0.001).

**Conclusion:**

The study highlights high patient satisfaction during the pandemic. Improving accessibility and quality of primary healthcare and community centres is essential to meet patient needs effectively.

**Supplementary Information:**

The online version contains supplementary material available at 10.1186/s12889-024-18986-w.

## Aims and objectives

This study aims to comprehensively assess patient satisfaction with healthcare services in Jammu & Kashmir during the Covid-19 pandemic. Specifically, the goals of the study are to:

• Evaluate patient satisfaction levels with medical care received during the pandemic, encompassing various aspects such as communication with healthcare providers, access to services, and overall care quality.
• Identify factors influencing patient satisfaction and dissatisfaction with healthcare services, including demographic characteristics, healthcare utilization patterns, and perceptions of healthcare delivery.
• Investigate the association between socio-demographic factors and health-related behaviors, such as alcohol and tobacco use, vaccination uptake, and healthcare seeking behaviors, to understand their impact on patient satisfaction and healthcare outcomes.

**Supplementary Information:**

The online version contains supplementary material available at 10.1186/s12889-024-18986-w.

## Introduction

Healthcare in India during the Covid-19 pandemic has undergone significant challenges, with the Indian government implementing a state-wide lockdown in March 2020 despite relatively low case numbers. This precautionary measure disrupted ordinary healthcare services [1, 2]. The pandemic prompted a major shift in hospital operations, transitioning inpatient care to outpatient settings and outpatient care to telemedicine-driven home care. As a result, in-person consultations decreased while online consultations surged [2].

The World Health Organization emphasizes the importance of health systems being responsive to patient and community needs [3]. Patient satisfaction is a crucial indicator of healthcare quality, as satisfied patients are more likely to adhere to treatment and utilize health services effectively [4, 5]. However, basic healthcare services in India have faced significant obstacles, leading to unsatisfactory outcomes for many residents [6].

The northern state of Jammu & Kashmir, in particular, has faced immense challenges in providing quality healthcare due to its poor healthcare infrastructure [7]. During the second wave of the pandemic, the region witnessed a high number of Covid-19 cases and deaths, further straining healthcare resources [8, 9]. Overwhelmed hospitals and understaffed healthcare centers resulted in prolonged wait times and compromised patient care [10].

Amidst these challenges, assessing healthcare delivery efficacy becomes paramount. The Covid-19 pandemic has underscored the importance of evaluating healthcare quality in India, particularly through the lens of patient satisfaction [11]. Patient satisfaction is a multifaceted concept encompassing various aspects of the healthcare experience, including communication with healthcare professionals, access to services, and overall care quality [12, 13].

However, there is limited research on the impact of Covid-19 on healthcare delivery in Jammu & Kashmir, an area characterized by socio-political complexities and limited healthcare infrastructure [14, 15]. Understanding how the pandemic has influenced healthcare delivery in conflict-affected regions like Jammu & Kashmir is crucial for informing policy and improving healthcare systems in such areas [16].

This study aims to fill this gap by investigating patient satisfaction with healthcare services in Jammu & Kashmir during the Covid-19 pandemic. By examining patient satisfaction levels during the Covid-19 pandemic, particularly in conflict-affected regions like Jammu & Kashmir, the study provides valuable insights into the effectiveness of healthcare delivery adaptations in response to global health crises.

## Methodology

This was a cross-sectional survey carried out among 277 participants in Jammu and Kashmir. We conducted a study about patient satisfaction on a large population affected by COVID-19, which includes factors such as epidemiology, demographics, and socio-behavioural changes.

**Study Period-** May 2023- August 2023.

### Method of data collection

#### The patient satisfaction questionnaire-18 (PSQ‐18)

Patient satisfaction was evaluated by using an adapted short version of the Patient Satisfaction Questionnaire (PSQ-18, Marshall and Hays) [17]. The questionnaire consists of 18 closed-type questions and is used for the evaluation of patients’ satisfaction with medical services in six main domains: General Satisfaction, Technical Quality, Interpersonal Manner, Communication, Financial Aspects, Time Spent with the Doctor, and Accessibility and Convenience. We also evaluated the total sum score of all subscales. This questionnaire employs a 5-point Likert scale, having scores as “Strongly Agree = 1, Agree = 2, Uncertain = 3, Disagree = 4, Strongly Disagree = 5”. Respondents received from 1 to 5 points for each answer, where 5 meant the highest satisfaction. According to the PSQ-18 scoring system, the sum score of all subscales may range from 18 to 90 points, where 18 points is the poorest possible evaluation and 90 points the best. To evaluate the internal consistency of the questionnaire, Cronbach’s alpha coefficient (0.96) was calculated and was found to be very good.

A pilot study was conducted with 130 participants to understand the trend of the region, their response to emergency and to ease the selection of participants (inclusion and exclusion). Before collecting data, each participant provided informed consent. Data was collected for the participants who were tested positive or were hospitalised due to Covid-19. The data collection was held in 2 stages. In the first stage, the purpose of the study was stated as following: “A survey was conducted to gauge patient satisfaction during the COVID-19 pandemic,” along with informed agreement, which was obtained prior to the data collection.

**Fig. 1:**
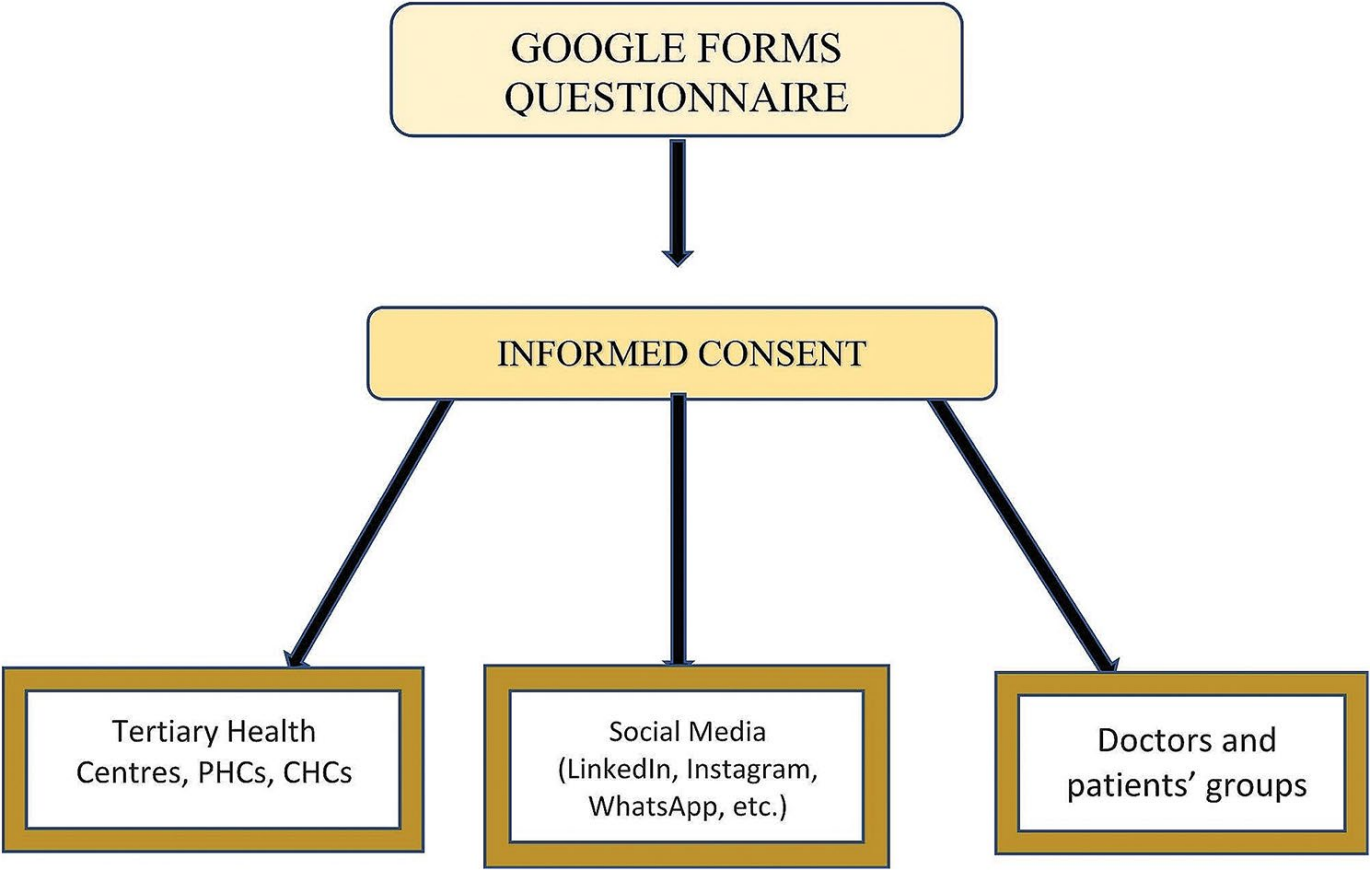
Mode of data collection

After the consent of the participants, the second stage of the data collection was conducted, where the questionnaire was distributed among Tertiary Health Centres, PHCs, CHCs, Social media channels and to doctors (Fig. 1). The questionnaire was filled by the participants. All the details obtained from the participants were systematically documented on Microsoft Excel.

### Sample size calculation

A study was conducted to determine the Patient Satisfaction Survey Of Covid- 19 Survivors. The research involved a sample size of 277 cases, which was chosen with a 95% confidence level and a margin of error of ± 12%.

The sample size was determined using the following formula: **n = z**^**2**^**p(1-p)/ d**^**2**^.

where:

**Z represents the z statistic at a 5% level of significance.**

**d is the margin of error.**

**p represents the expected prevalence, which was set at 31%.**

#### Sampling method

The Sampling Method used was Simple random sampling. We utilized a simple random sampling method, which is a type of probability sampling technique where every individual in the population has an equal chance of being selected for the study. This method ensures that the sample is representative of the larger population and reduces the risk of bias in participant selection.

To determine the sample size, we conducted a study to determine the Patient Satisfaction Survey Of Covid-19 Survivors, choosing a sample size of 277 cases. This sample size was calculated with a 95% confidence level and a margin of error of ± 12%. The formula used for sample size calculation was n = z^2 * p * (1-p) / d^2, where ‘n’ represents the sample size, ‘z’ is the z statistic at a 5% level of significance, ‘p’ is the expected prevalence, and ‘d’ is the margin of error. In our study, we set the expected prevalence at 31%.

Once the sample size was determined, we selected participants randomly from the population of COVID-19 survivors in Jammu and Kashmir. This approach ensured that every individual had an equal opportunity to be included in the study, enhancing the generalizability of our findings to the broader population (Table 1).

**Table 1:** Inclusion criteria

Inclusion criteria	
1.	Those participants who gave informed consent for the study.
2.	Those participants who were hospitalized for COVID-19 treatment and were discharged after treatment.
3.	Only the participants from Jammu and Kashmir were included.
4.	Both genders and all age groups above 18 years of age at the time of admission

It is important to note that simple random sampling is a widely accepted and rigorous method for selecting participants in research studies, as it helps minimize selection bias and ensures that the sample is representative of the population of interest. By employing this sampling strategy, we aimed to obtain reliable and valid data on patient satisfaction and healthcare delivery amidst the COVID-19 pandemic in Jammu and Kashmir.

### Statistical analysis

The data collected from the respondents was initially entered into MS Excel spreadsheets and categorized as well as tabulated using Microsoft Excel (version 2009). The statistical software SPSS (version 25) was used for analysing the data. Chi-Square test and Descriptive analysis was performed.

All experimental protocols were approved by **University of Amity Institutional Review Board (IRB 330 No. AUUP/IEC/MAY/2023/4)**.

Informed consent was obtained from all subjects and/or their legal guardian(s).

## Results

### Socio- demographic profile

In this study, the final sample size composed of 277 patients who participated and completed the survey. Of these participants, 86.3% were males, while females accounted for 13.7%. Table 2. shows that the majority of the study’s population were between 18 and 33 years old.

**Table 2:** Socio demographic profile of respondents

Variables		Number (%)	(SD)
Age	18–33 years	137 (49.5)	≈ 12.97 years
	34–47 years	88 (31.8)	
	48 and above years	52 (18.8)	
Gender	Male	239 (86.3)	≈ 0.344
	Female	38 (13.7)	
Working sector	Private	97 (35)	≈ 0.477
	Government	165 (59.6)	≈ 0.49
	Self- Employed	15 (5.4)	≈ 0.226
Religion	Hindu	191 (69)	≈ 0.463.
	Muslim	86 (31)	
Education	Uneducated/ Illiterate	10 (3.6)	≈ 27.96
	Secondary	13 (4.7)	
	Higher Secondary	41 (14.8)	
	Graduate	199 (71.8)	
	Post Graduate	14 (5.1)	
Marital status	Unmarried	58 (22.6)	≈ 0.449
	Married	199 (77.4)	

It was observed that nearly 35% of the individuals who were surveyed worked in the private sector, around 60% were employed in the government sector and only 15% of respondents reported being self-employed. As per the education qualifications, majority of the participants were Graduates (71.8%).

Additionally, the study revealed that only a small proportion of individuals, around 4%, reported being either uneducated or illiterate. A significant number of the participants, around 69%, identified as Hindu, while only 31% reported being Muslim.

### Factors affecting patient satisfaction

#### Factors associated with patient satisfaction (Agreement reflecting satisfaction with medical care)

**Fig. 2:**
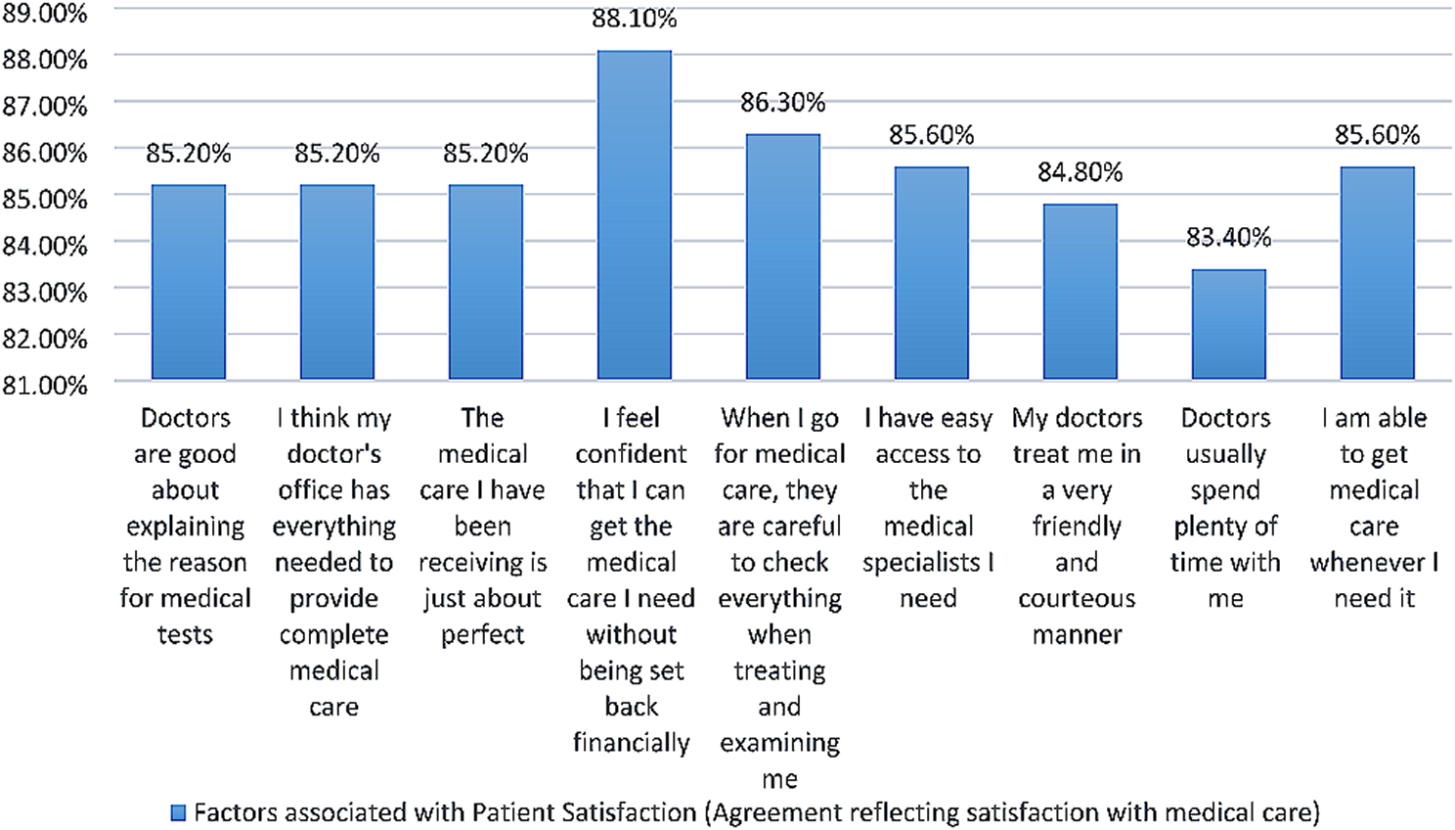
Factors associated with patient satisfaction (agreement reflecting satisfaction with medical care)

Table 3. and Fig. 2, depicts that the majority of the participants expressed a high level of satisfaction with the medical care they received (85.2%). In terms of doctors’ explanations of medical tests (85.2%), majority of respondents agreed that their doctors were good about explaining the reason for medical tests. Additionally, 85% participants strongly agreed that their doctor’s office had everything needed to provide complete medical care, and that the medical care they had been receiving was just about perfect.

**Table 3:** Factors associated with patient satisfaction (agreement reflecting satisfaction with medical care)

Cariable	Strongly Agree	Agree	Uncertain	Disagree	Strongly Disagree
1. Doctors are good about explaining the reason for medical tests	236 (85.2%)	10 (3.6%)	11 (4%)	15 (5.4%)	5 (1.8%)
2. I think my doctor’s office has everything needed to provide complete medical care	236 (85.2%)	10 (3.6%)	12 (4.3%)	14 (5.1%)	5 (1.8%)
3. The medical care I have been receiving is just about perfect	237 (85.2%)	6 (2.2%)	12 (4.3%)	17 (6.1%)	5 (1.8%)
4. I feel confident that I can get the medical care I need without being set back financially	244 (88.1%)	7 (2.5%)	11 (4%)	13 (4.7%)	2 (0.7%)
5. When I go for medical care, they are careful to check everything when treating and examining me	239 (86.3%)	6 (2.2%)	14 (5.1%)	13 (4.7%)	5 (1.8%)
6. I have easy access to the medical specialists I need	237 (85.6%)	9 (3.2%)	10 (3.6%)	16 (5.8%)	5 (1.8%)
7. My doctors treat me in a very friendly and courteous manner	235 (84.8%)	14 (5.1%)	12 (4.3%)	13 (4.7%)	3 (1.1%)
8. Doctors usually spend plenty of time with me	231 (83.4%)	11 (4%)	15 (5.4%)	16 (5.8%)	4 (1.4%)
9. I am able to get medical care whenever I need it	237 (85.6%)	6 (2.2%)	12 (4.3%)	17 (6.1%)	5 (1.8%)

*Cronbach’s alpha (0.96)

In terms of financial accessibility, 88% respondents indicated that they felt confident they could get the medical care they needed without being set back financially. Similarly, when it came to medical examinations, majority of participants felt that their doctors were careful to check everything when treating and examining them.

Furthermore, 85% respondents reported that they had easy access to the medical specialists they needed, and that their doctors treated them in a very friendly and courteous manner. Only 1.4% of participants didn’t agreed that their doctors spent plenty of time with them during appointments.

Overall, the research findings suggest that the majority of patients surveyed were highly satisfied with their medical care experiences, with many expressing strong agreement with these factors associated with patient satisfaction.

#### Factors associated with patient satisfaction (agreement reflects dissatisfaction with medical care)

**Fig. 3:**
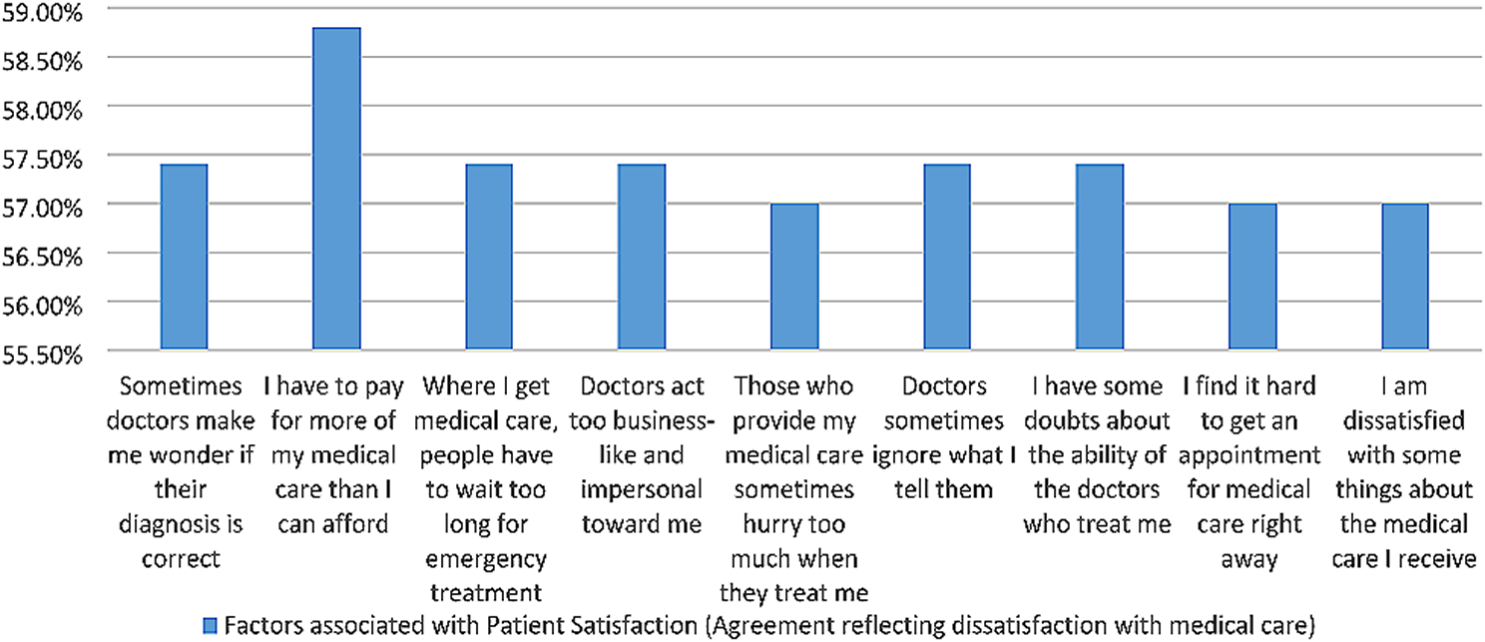
Factors associated with patient satisfaction (agreement reflecting dissatisfaction with medical care)

According to Table 4. And Fig. 3, participants surveyed in this study reported high levels of disagreement with the factors associated with patient satisfaction reflecting dissatisfaction. Majority of participants disagreed that doctors sometimes made them wonder if their diagnosis was correct (57.4%), and that doctors sometimes ignored what they told them (76.2).

**Table 4:** Factors associated with patient satisfaction (agreement reflecting dissatisfaction with medical care)

Variable	Strongly Agree	Agree	Uncertain	Disagree	Strongly Disagree
10. Sometimes doctors make me wonder if their diagnosis is correct	15 (5.4%)	6 (2.2%)	11 (4%)	86 (31%)	159 (57.4%)
11. I have to pay for more of my medical care than I can afford	4 (1.4%)	1 (0.4%)	9 (3.2%)	100 (36.1%)	163 (58.8%)
12. Where I get medical care, people have to wait too long for emergency treatment	21 (7.6%)	9 (3.2%)	4 (1.4%)	84 (30.3%)	159 (57.4%)
13. Doctors act too business-like and impersonal toward me	20 (7.2%)	5 (1.8%)	3 (1.1%)	159 (57.4%)	90 (32.5%)
14. Those who provide my medical care sometimes hurry too much when they treat me	21 (7.6%)	9 (3.2%)	5 (1.8%)	84 (30.3%)	158 (57%)
15. Doctors sometimes ignore what I tell them	20 (7.2%)	6 (2.2%)	4 (1.4%)	88 (31.8%)	159 (57.4%)
16. I have some doubts about the ability of the doctors who treat me	14 (5.1%)	6 (2.2%)	9 (3.2%)	89 (32.1%)	159 (57.4%)
17. I find it hard to get an appointment for medical care right away	16 (5.8%)	5 (1.8%)	10 (3.6%)	88 (31.8%)	158 (57%)
18. I am dissatisfied with some things about the medical care I receive	16 (5.8%)	9 (3.2%)	11 (4%)	83 (30%)	158 (57%)

*Cronbach’s alpha (0.96)

Major respondents also disagreed with the assertion that they had doubts about the ability of the doctors who treated them (57.4%), and around 57% participants mentioned that the doctors or healthcare providers hurried too much when they were treating them with medical care.

Furthermore, 58.8% disagreed that they had to pay for more of their medical care than they could afford, and that where they received medical care, people had to wait too long for emergency treatment (57.4%).

57.4% respondents also disagreed with the fact that doctors acted too business-like and impersonal toward them, and that they found it hard to get an appointment for medical care right away.

Overall, the research findings suggest that the majority of patients surveyed were highly satisfied with their medical care experiences, with many expressing strong disagreement reflecting dissatisfaction with these factors associated with patient satisfaction.

This indicates that healthcare providers may be doing a good job of meeting the needs and expectations of their patients, and that patients are generally satisfied with the quality of care they receive.

#### Calculating the patient satisfaction level using patient satisfaction scale (PSQ-18)

Patient Satisfaction Questionnaire Short Form (PSQ-18), a concise, validated tool that is applied to various settings, as well as comparing interventions. The PSQ-18 is an established questionnaire that is widely used across the world for measuring patient satisfaction levels.

To calculate the score: To (Table 5.)

**Table 5:** Scoring items

Item Number (Question)	Original Response Value	Scored Value
1,2,3,4,5,6,7,8,9 10,11,12,13,14,15,16,17,18

- for factors associated with patient satisfaction (Agreement reflecting satisfaction with medical care)
  Total score = 45
- for factors associated with patient satisfaction (Agreement reflecting dissatisfaction with medical care)
  Total score = 44
  Grand total = 45 + 44 = 89

**The Patient satisfaction score (89) hence proved that there was significantly higher level of satisfaction among patients regarding medical care.**

### Association between socio demographic factors and different variables

We conducted a chi-square analysis (Table 6.) to test the hypothesis that there is a relationship between gender and use of alcohol. The analysis revealed an uncertainty associated with borderline results between gender and use of alcohol *(p* value = 0.047), with emphasis on the need for further research to confirm or refute the findings, and showed significant association with gender and use of tobacco *(p value = 0.000)*.

**Table 6:** Association between socio demographic factors and different factors

Sno.	Variables		Vaccination Yes no (*N*%) (*N*%)		*P*-VALUE (*P* > 0.05)	Use of alcohol Yes no (*N*%) (*N*%)		*P*-Value (*P* > 0.05)	Use of tobacco Yes no (*N*%) (*N*%)		*P*-Value (*P* > 0.05)
1.	Age	18–33 Yrs	132	5	0.334	28	109	0.402	50	87	0.607
		34–47 Yrs	82	6		20	68		36	52	
		48- Above	51	1		7	45		17	35	
2.	Gender	Male	228	11	0.579	52	187	**0.047**	100	139	**0.000**
		Female	37	1		3	35		3	35	
3.	Marital status	Married	55	3	0.836	3	55	**0.001**	17	41	0.216
		Unmarried	190	9		48	151		76	123	
4.	Education	Uneducated	10	0	0.190	1	9	0.083	3	7	0.692
		Secondary	11	2		2	11		5	8	
		Higher secondary	38	3		4	37		19	22	
		Graduate	192	7		42	157		70	129	
		Post graduate	14	0		6	8		6	8	
5.	Religion	Muslim	82	4	0.861	5	81	**0.000**	39	47	0.059
		Hindu	183	8		50	141		64	127	
6.	Occupation	Student	40	1	0.506	7	34	0.766	11	30	**0.008**
		Others	204	10		43	171		90	124	
		Health/ police	14	0		4	10		2	12	
		None	7	1		1	7		0	8	

The values in bold signifiy statistically significant values

The analysis between marital status with use of alcohol, revealed a statistically significant association with *p value = 0.001*. There was also a significant association between religion and use of alcohol *(p value = 0.000)*. Use of tobacco and occupation showed a significant association with the *p value = 0.008.*

## Disscussion

The study findings revealed a high level of satisfaction among the majority of participants (85.2%) with the medical care they received, indicating a positive perception of healthcare services in the region. Participants acknowledged their doctors’ proficiency in explaining medical tests, the availability of necessary resources in doctors’ offices, and the quality of medical care received, reflecting positively on healthcare delivery. Moreover, participants expressed confidence in accessing medical care without financial constraints and perceived their doctors as thorough and attentive during examinations and treatments. Additionally, easy access to medical specialists and courteous treatment from doctors further contributed to overall satisfaction with healthcare services [18].

The analysis uncovered significant relationships between demographic factors and health-related behaviours. Specifically, there was a notable association between age and alcohol use, suggesting a potential correlation between increasing age and higher likelihood of alcohol consumption. Furthermore, gender was found to be associated with both alcohol and tobacco use, highlighting differences in usage patterns between males and females. Additionally, the significant association between education level and vaccination uptake suggests that educational attainment may influence individuals’ decisions regarding vaccination.

The unique context of Jammu and Kashmir, characterized by its socio-political situation and limited healthcare infrastructure, underscores the significance of this research. The region’s ongoing conflicts and political instability present significant challenges for the healthcare system, exacerbating the difficulties of managing the COVID-19 pandemic [19]. Understanding the impact of COVID-19 on healthcare outcomes in such conflict-affected regions is crucial for informing effective healthcare policies and interventions.

Practical implications of these findings include the importance of tailored interventions to address age-specific alcohol use patterns, gender-sensitive approaches to tobacco cessation programs, and targeted educational campaigns to promote vaccination uptake. Additionally, efforts to strengthen healthcare infrastructure and improve access to medical services are essential for mitigating the impact of the COVID-19 pandemic in conflict-affected areas like Jammu and Kashmir [20].

However, it is important to acknowledge the limitations of the study, such as its cross-sectional design and potential for response bias. Future research should consider longitudinal studies and explore additional factors influencing healthcare outcomes in conflict-affected regions. Overall, the study provides valuable insights into the complexities of healthcare delivery in challenging socio-political contexts and underscores the importance of addressing these issues to improve health outcomes for all.

## Conclusion

The study findings indicate that patients expressed high levels of satisfaction during the pandemic era. To improve patient satisfaction, there is a need for more primary healthcare centres and community health centres. These facilities play a crucial role in providing accessible and affordable healthcare services to the community. By expanding the reach of these centres and improving their quality of care, patients can receive the care they need in a timely and effective manner. Additionally, by addressing the specific needs of each patient, these centres can help ensure that patients are satisfied with their healthcare experience, leading to better health outcomes and overall satisfaction.

Patient satisfaction is an important aspect of healthcare delivery, and it has been shown to be associated with better clinical outcomes and improved adherence to treatment plans. In India, there is a need for further research to identify the factors that contribute to patient satisfaction and to develop interventions that can improve it. This is particularly important in light of the challenges that the healthcare system in India faces, including limited resources, high patient volumes, and disparities in access to care. Future research could focus on areas such as patient-provider communication, quality of care, and access to healthcare services. By addressing these issues, healthcare providers and policymakers can work towards improving patient satisfaction and ultimately enhancing the overall quality of healthcare in India.

The Covid-19 pandemic has disrupted healthcare systems worldwide, prompting significant changes in service delivery, patient-provider interactions, and healthcare utilization patterns. Understanding how these changes have influenced patient satisfaction and healthcare outcomes is crucial for informing pandemic response strategies and building resilient healthcare systems internationally.

### Limitations

- The study is confined to only one State i.e. Jammu and Kashmir, so to know the level of satisfaction on a larger level we need to conduct further research in this area. The study utilized a sample from Jammu and Kashmir, which may not be representative of the broader population of India. The findings might not accurately reflect the diversity of healthcare experiences across different regions and socio-economic groups within the country.
- Selection Bias: The study only included COVID-19 survivors who were hospitalized, excluding those who were not hospitalized or received treatment outside hospitals. This exclusion could skew the results towards individuals with more severe cases, potentially overlooking the satisfaction levels of asymptomatic or mildly affected individuals.
- The cross-sectional design provides a snapshot of patient satisfaction at a single point in time. It does not capture changes or trends in satisfaction over time, limiting the understanding of long-term healthcare delivery dynamics.
- We have taken only COVID-19 survivors as our target population to know the patient satisfaction level, so we didn’t include the ones who were not hospitalised and consulted a doctor offline or virtually.

### Recommendations

- The need for more resources in the area of patient satisfaction stems from the importance of quality healthcare for patients. Patients who are satisfied with their healthcare experience are more likely to comply with treatment, keep follow-up appointments, and utilize health services.
- “Quality of healthcare matters”- It highlights the importance of considering the perspectives and needs of both the patient and healthcare provider. Both parties play an equally important role in ensuring quality healthcare delivery.
- Assessing patient satisfaction can be a novel approach for further novel epidemic/pandemic because it provides valuable insights into the effectiveness of the healthcare system in dealing with such crises.
- The need for preparedness is critical for any country to effectively respond to any crisis, including natural disasters, pandemics, and other emergencies. This preparedness involves having a robust healthcare system, equipped with adequate resources, facilities, and healthcare professionals to handle any emergency situation.

### Electronic supplementary material

Below is the link to the electronic supplementary material.

Supplementary Material 1

## Data Availability

The datasets used and/or analysed during the current study are available from the corresponding author on reasonable request.
