# Supplementary material for: Assessing patient satisfaction and healthcare delivery amidst the COVID-19 pandemic: insights from Jammu and Kashmir, India
Source: BMC Public Health. 2024 Aug 1;24:2082. doi: 10.1186/s12889-024-18986-w (PMC11295500; doi:10.1186/s12889-024-18986-w)
Supplement: Supplementary file 1 — Supplementary Material 1 [file 12889_2024_18986_MOESM1_ESM.pdf]

SHORT-FORM PATIENT SATISFACTION QUESTIONNAIRE (PSQ-18)

These next questions are about how you feel about the medical care you receive.

On the following pages are some things people say about medical care. Please read each one carefully, keeping in mind the medical care you are receiving now. (If you have not received care recently, think about what you would expect if you needed care today.) We are interested in your feelings, good and bad, about the medical care you have received.

How strongly do you AGREE or DISAGREE with each of the following statements?

(Circle One Number on Each Line)

	Strongly Agree	Agree	Uncertain	Disagree	Strongly Disagree
1. Doctors are good about explaining the reason for medical tests	1	2	3	4	5
2. I think my doctor's office has everything needed to provide complete medical care	1	2	3	4	5
3. The medical care I have been receiving is just about perfect	1	2	3	4	5
4. Sometimes doctors make me wonder if their diagnosis is correct	1	2	3	4	5
5. I feel confident that I can get the medical care I need without being set back financially	1	2	3	4	5
6. When I go for medical care, they are careful to check everything when treating and examining me	1	2	3	4	5
7. I have to pay for more of my medical care than I can afford	1	2	3	4	5
8. I have easy access to the medical specialists I need	1	2	3	4	5

How strongly do you AGREE or DISAGREE with each of the following statements?

(Circle One Number on Each Line)

	Strongly Agree	Agree	Uncertain	Disagree	Strongly Disagree
9. Where I get medical care, people have to wait too long for emergency treatment	1	2	3	4	5
10. Doctors act too businesslike and impersonal toward me	1	2	3	4	5
11. My doctors treat me in a very friendly and courteous manner	1	2	3	4	5
12. Those who provide my medical care sometimes hurry too much when they treat me	1	2	3	4	5
13. Doctors sometimes ignore what I tell them	1	2	3	4	5
14. I have some doubts about the ability of the doctors who treat me	1	2	3	4	5
15. Doctors usually spend plenty of time with me	1	2	3	4	5
16. I find it hard to get an appointment for medical care right away	1	2	3	4	5
17. I am dissatisfied with some things about the medical care I receive	1	2	3	4	5
18. I am able to get medical care whenever I need it	1	2	3	4	5
